# Tomato yield, and water use efficiency as affected by nitrogen rate and irrigation regime in the central low lands of Ethiopia

**DOI:** 10.1038/s41598-024-62884-5

**Published:** 2024-06-10

**Authors:** Beza Shewangizaw, Kenzemed Kassie, Shawl Assefa, Getachew Lemma, Yalemegena Gete, Demisew Getu, Lisanu Getanh, Getanh Shegaw, Gebrehana Manaze

**Affiliations:** Amhara Regional Agricultural Research Institute, Debra Birhan Agricultural Research Center, Debra Birhan, Ethiopia

**Keywords:** Potential evapotranspiration from the crop (ETc), Yield, Nutrient, Water use efficiency, Environmental chemistry, Hydrology

## Abstract

Tomato yield can be increased by the application of optimum water and fertilizer. A field experiment was conducted in Efratana Gidim district, North Shewa, Amhara, Ethiopia, during 2019 and 2020. The objective was to determine the nitrogen (N) rate and irrigation regime for optimum tomato yield and water use efficiency (WUE). The experiment consisted of three-irrigation regimes (75% ETc (Evapotranspiration from the crop), 100% ETc, and 125% ETc) and four nitrogen (N) rates (control; i.e. without N application^1^, 46 kg N ha^−1^, 92 kg N ha^−1^, and 138 kg N ha^−1^). The treatments were laid out in a split-plot design with four replications. The Irrigation regime were assigned to the main plot, while the N rate were assigned to the subplot. Data on growth, yield, and yield-related traits of tomatoes, include; plant height, number of fruit clusters per plant, fruit length, fruit diameter, number of marketable fruits, number of un-marketable fruits, the total number of fruits, marketable fruit yield, un-marketable fruit yield, total yield were collected. The data were subjected to analysis of variance using R studio. The results indicated that the experimental site had low total N content, and the application of N fertilizer significantly improved tomato yield. Increasing irrigation depth also significantly increased tomato yield. The result indicated that the highest mean marketable fruit yield (35,903 kg ha^−1^) was obtained from the combined application of 125% ETc with 92 kg N ha^−1^, while the lowest (13,655 kg ha^−1^) marketable fruit yield was obtained from 75% ETc with 92 kg N ha^−1^. The analysis of variance showed that the highest (5.4 kg m^−3^) WUE recorded from 75% ETc with 46 kg N ha^−1^ increased WUE by 77% (2.4 kg m^−3^) compared with the lowest (2.3 kg m^−3^) WUE recorded from 125% ETc with 0 kg N ha^−1^. The partial budget analysis also indicated that the highest net benefit (266,272 ETB (Ethiopian Birr) ha^−1^) and an acceptable marginal rate of return (1240%) for the invested capital was recorded from the combined application of 125% ETc with 92 kg N ha^−1^. Therefore, the application of 125% ETc with 92 kg N ha^−1^ resulted in the highest net benefit.

## Introduction

The Tomato (*Solanum lycopersicum*) is the most widely grown vegetable in the world. The crop is a source of vitamins, minerals and antioxidants, which are important for human diets. The crop also contains lycopene, which is responsible for reducing different cancers and neurodegenerative diseases^1^ Ethiopia has an enormous potential for tomatoes production due to several attributed that are favorable to their growth, such as soil, climate and topography^2^. The crop is one of the most profitable crop, providing a higher income for farmers. According to FAO^3^, the production of Tomato is estimated to be 55,000 tons in 2013 but showed a decreasing trend compared with the production recorded in 2011 (81,738 tons). The possible reason attributed to irrigation water, disease and pest (such as tutaabsoluta and late blight), poor agronomic practice, shortage of improved varieties, poor quality seed and post-harvest handling practice^4–7^. Nutrients, especially nitrogen and Phosphorus, can be the major limiting factor for plant growth and development next to sunlight and water^8,9^. Nitrogen is essential for building up of protoplasm and protein, which is responsible for cell division and initial meristematic activity (Singh and Kumer 1996). It also promotes flower and fruit setting of tomatoes. Thus, nitrogen has a positive effect on tomato growth and development in soil with limited N supplies^10^. Next to nitrogen fertilizer, phosphorus containing fertilizers are the second most important input for increasing crop production. A high level of phosphorus throughout the root zone is essential for rapid root development and for good utilization of water and other nutrients by the plant. Tomatoes have the greatest demand for phosphorus at the early stages of development^11,12^.

In Ethiopia, fertilizer rates, especially N and P were determined for tomatoes in some parts of Ethiopia. But the rate, for instance, the fertilizer recommendation for N ranged between 56 and 230 kg ha^−1^ and for P ranged from 48 to 137 kg ha^−1^^13–15^. The probable reason for this attributed to soil and agro-ecological variability. Moreover, these recommendations were too general to use for specific areas. Urea is a type of nitrogen fertilizer that can be used for tomato plants. Urea has some advantages over other forms of nitrogen fertilizer, such as: It is easy to handle, store and apply, it has a high nutrient analysis (46% nitrogen) and a reasonable price, it poses no explosion hazards, unlike ammonium nitrate, it can increase crop yields by a great amount, especially when applied through drip irrigation, and It can enhance the metabolism and growth rate of plants^16^.

Ethiopia's rain fed agriculture is a major contributor to both the country's overall economy and food supply. Because of this, the prosperity of agriculture has been heavily driven by rainfall availability. Given the country’s highly variable rainfall patterns, the unreliability of rainfall has negatively affected Ethiopia’s economy in general and its agriculture sector in particular. Water scarcity, combined with low use of improved farm inputs, decreases crop yields, which leads to low crop production, food insecurity, and poverty^17^. Therefore, the expansion of irrigation is essential to reducing crop failure risk and maintaining agricultural output^18,19^. Ethiopia has 5.3 million hectares of potential for irrigation, of which 3.7 million hectares can be developed using surface water sources and 1.6 million hectares with groundwater and rain water management^18,20–22^, over which Over half of this is categorized under small-scale, which are often characterized by low water productivity^23,24^. Ethiopia began using conventional irrigation techniques in the 1950s. Nowadays, several regions of the nation’s use modern irrigation techniques like drip and sprinkler irrigation^18,25^. Since irrigation uses a large volume of water that is taken from many sources, effective water management and utilization are the primary challenges^25^.

Many irrigation systems in Ethiopia including Eferatagidm for most crops including tomatoes are traditional and not supported by research findings^26^. Therefore, the farmers had a low awareness of irrigation water management^27^. As the result, most farmers do not have adequate facilities, knowledge, tools and skills to manage irrigation water. Some of the common challenges faced by farmers are: over or under irrigation, lack of irrigation scheduling for most crops, poor irrigation infrastructure and inadequate water governance^25,27,28^. Combined management of irrigation water and soil fertility can increase the yield of tomatoes and the percentage of water saving that can be utilized to irrigate more area^27,29–31^. Both under and over application of water to the tomato crop had a negative impact. Under application of water can reduce photosynthesis, transpiration, stomatal conductance, leading to lower carbon assimilation and water use efficiency. Which also impaired nutrient uptake and transport, especially calcium, which can cause physiological disorders such as blossom-end rot, growth cracks, and sun scald in the fruits^32,33^. Too much water can be detrimental to tomato plants, as it can cause a number of problems, such as: root rot, blossom end rot, fruit cracking, and also resulted in sun scald. Therefore, tomato plant need to be well irrigated^34^. Therefore, optimum application of irrigation water is mandatory^35^.

In collaboration with AGP (Agricultural Growth program) our research center conducted production constraint assessment on AGP supported district Eferatagidm. The result of the assessment indicated that; Onion and tomato were the most important vegetable crops and there was no fertilizer and irrigation water recommendation for these crops. Thus, the present study was implemented with the objectives of determining nitrogen (N) rate and irrigation regime for optimum tomato yield, water use efficiency, and economic return in Eferatagidm district and similar areas.

## Materials and methods

### Description of the study area

**Figure 1:**
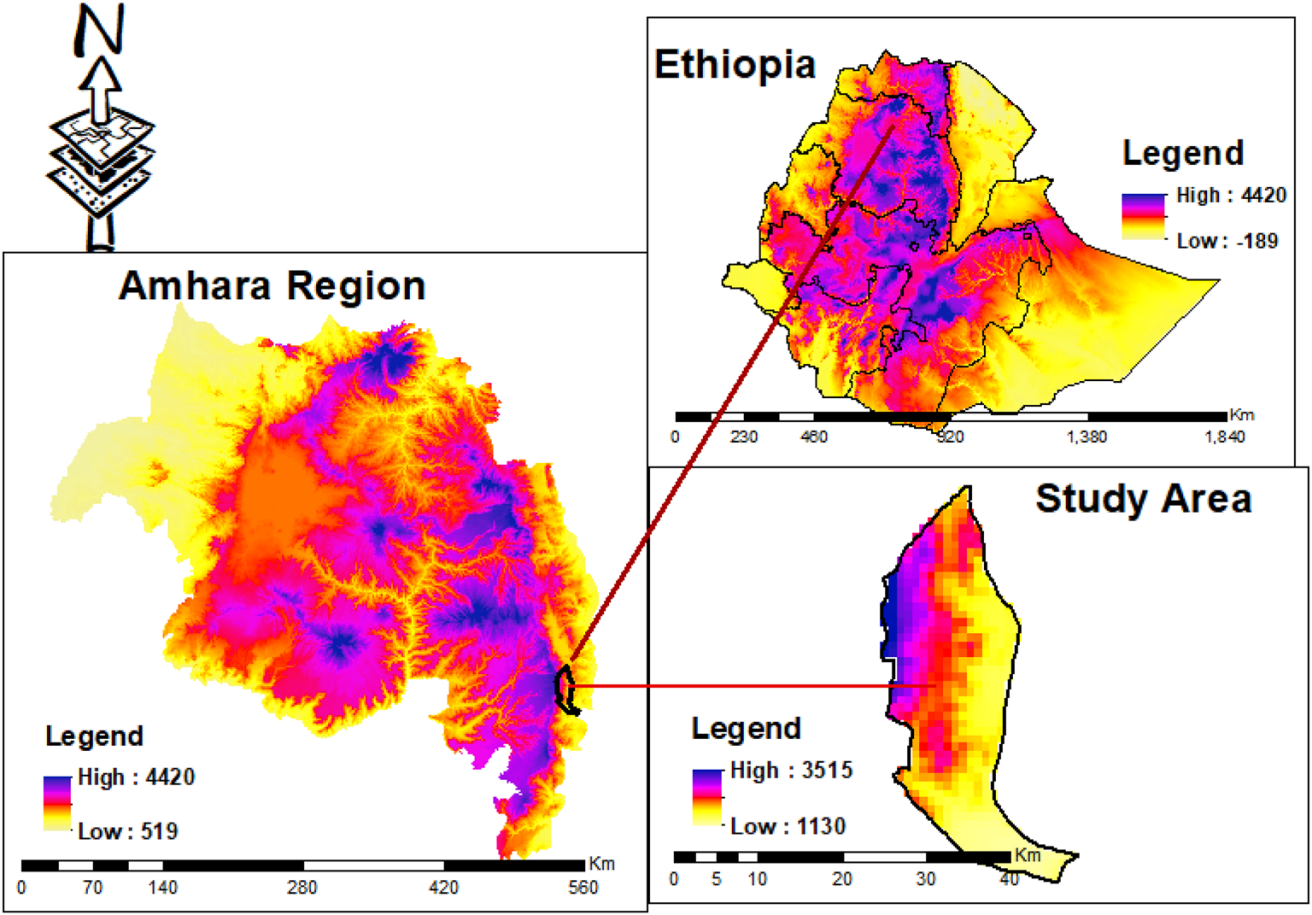
Location map of the study area.

**Figure 2:**
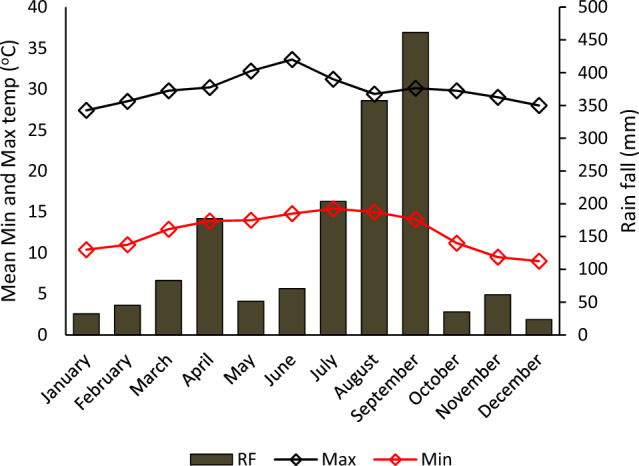
Long-term metrological data of the experimental field.

The experiment was conducted in the Efratana Gidm district, located in the North Shewa Zone of the Amhara Regional State, during the irrigation season of 2019 and 2020. The Efratana Gidm district lies between 10° 5′ N–100 32′N and 39° 50′ E– 390 0′ E latitude and longitude (Fig. 1). The altitude ranges from 1130 to 3515 m above sea level (masl) (Fig. 1). The topography of the district is generally rugged and broken, with many hills and ridges, making most part of the area unsuitable for agriculture, even though cultivated. The district has minimum and maximum average annual temperatures of 12.6 °C and 29 °C, respectively. The average 40 years’ annual rainfall is 1177.14 mm (Fig. 2). The major land use pattern of the district includes croplands 47%, forest and bush 23%, and grazing 10%. The district is well known for its underground and surface water like rivers and streams. Nazero, Jewuha and Jara are the three big rivers known in the district. The dominant crops cultivated in the district are sorghum (*Sorghum bicolor)*, tef (*Eragrostis tef*), maize (*Zea mays*), mung bean (*Vigna radiata*), haricot bean (*Phaseolus vulgaris)*, onion (*Allium cepa)* and tomato (*Solanum lycopersicum)*. Disease and pest, lack of access to improved technologies, shortage of post-harvest handling techniques of onion and tomato, and lack of fertilizer recommendations are some of the challenges for crop production in the district^4,36,37^. The long term rain fall, maximum and minimum temperature of the district were presented in Fig. 2.

### Treatment, design, and experimental procedure

The treatments consisted of four levels of nitrogen (Control; i.e. without N application, 46, 92 and 138 kg N ha^−1^) and three levels of irrigation regime expressed as a percentage of potential Evapotranspiration (ETc), i.e., IRR1 (75% ETc), IRR2 (100% ETc) and IRR3 (125% ETc). The base for selecting nitrogen rate was derived from the previous nitrogen recommendation and to align the recommendation with the current fertilizer label (46 kg N with 100 kg urea). The experiment was laid out in a split plot design with four replications. The main plot was assigned to the irrigation regime, while the sub-plot was assigned to the nitrogen rate. The experimental field was prepared following the conventional tillage practice before planting. The space between blocks and plots were 1.5 and 1 m, respectively. Ridges were constructed between block and plot to control movement of water and fertilizer from one plot to the other. The gross plot size for the main plot was 14.1 * 4 m (56.4 m^2^) and for the sub-plot was 2.4 * 4 m (9.6 m^2^), which is 4 rows and 8 plants per row. The harvestable plot size was 2 * 2.4 (4.8 m^2^).

The tomato varieties *Kochero* and *Weyno* were used as a test crop for the first and second year of the experiment, respectively. The reason for the varietal difference was attributed to the unavailability of Kochero variety seeds in the market. The seeds were sourced from DBARC (Debra Birhan Agricultural Research Center), horticulture research case team. Seedlings were grown on a seedbed for 1 month. The seedlings were supplied with nitrogen nutrient from urea. Uniform seedlings were transplanted and planted to the prepared ridges with spacing of 30 cm and 100 cm for plants and rows, respectively. The irrigation depth and frequency for each growing season were applied using FAO CROPWAT 8.1 model (Table 1). Then, the required amounts of irrigation water applied for each treatment were calculated by multiplying the depth of irrigation water with the area of the plots. The water was applied using the cane method. The depths of effective rainfall during the growth period (collecting on site by installing rain gauge) were deducted from the depth of irrigation water for the respective growth period (Table 2). Disease and pest were regularly monitored, and treatment were applied based on the recommendations of research.

**Table 1:** Depth of irrigation water during the growth period.

Month	Growth period	Depth of irrigation water (mm)		
		75% ETC	100% ETC	125% ETC
January	Initial	22.3	29.7	37.1
January	Initial	22.3	29.7	37.1
January	Initial	22.3	29.7	37.1
January	Initial	22.3	29.7	37.1
January/February	Developmental	22.3	29.7	37.1
February	Developmental	40.1	53.4	66.8
February	Developmental	40.1	53.4	66.8
March	Mid	62.3	83.1	103.9
March	Mid	62.3	83.1	103.9
April	Late	62.3	83.1	103.9
April	Late	62.3	83.1	103.9
Total irrigation depth (mm)		440.8	587.7	734.6
Total irrigation water (m^3^ ha^−1^)		4407.9	5877.2	7346.4

**Table 2:** Effective rainfall recorded during the growth period.

Year	Date	Growth stage	Effective rainfall (mm)	Year	Date	Growth stage	Effective rainfall (mm)
1	April 3, 2019	Late	5	2	April 6, 2020	Late	18
1	April 4, 2019	Late	10	2	April 10, 202	Late	12
1	April 5, 2019	Late	30	2	April 11, 2020	Late	9
1	April 8, 2019	Late	14	2	April 15, 2020	Late	24
1	April 14, 2019	Late	2	2	April 18, 2020	Late	14
1	April 15, 2019	Late	5	2	April 21, 2020	Late	8
1	April 16, 2019	Late	14	2	April 22, 2020	Late	31
1	April 17, 2019	Late	35	2	April 27, 2020	Late	18
1	April 21, 2019	Late	7	2	April 29, 2020	Late	8
1	April 17, 2019	Late	3				
1	April 30, 2019	Late	18				

An equal amount of phosphorus (40 kg P ha^−1^) was applied to all plots at planting from TSP. Nitrogen from urea was applied in split half at planting and the rest half after 45 days after transplanting the seedlings. A rain gauge was installed in the experimental field to collect rainfall data. The rainfall (effective rainfall) was deducted from the amount of irrigation water applied when it occurs in the irrigation interval. In 2019 and 2020 effective rainfall were recorded in a total of 11 and 9 days, respectively (Table 2). The long-term meteorological data of the station also indicated that the experimental area received 177.5 mm rain during these years (Fig. 2). The field performances of the experiment were presented in Figs. 4 and 5.

### Soil sampling and analysis

Composite surface soil samples were collected from a depth of 0–20 cm before planting for the determination of soil physicochemical properties. The samples were air dried, ground, and passed through a 2 mm sieve for most parameters, except for organic carbon (OC) and total nitrogen, which passed through a 0.5 mm sieve. The soil texture was determined by hydrometer method^38^. Soil pH was measured with a digital pH meter potentiometerically in supernatant suspension of 1:2.5 soil to distilled water ratio^39^. The cation exchange capacity (CEC) was determined by a 1 M ammonium acetate method at pH 7^40^, while organic carbon (OC) was determined by the dichromate oxidation method^41^. Total N in the soil was measured by the micro kjeldhal method^42^. Available phosphorus was analyzed by the Olsen method^43^ and measured colorimetrically by the ascorbic acid- molybdate blue method^44^.

### Data collection

The following data were collected at different growth stages of tomato, following standard guidelines for research in Ethiopia^45^:

- *Plant height (cm)* Ten plants were selected randomly from each experimental plot to measure plant height by a steel tape from the ground to the main apex during 50% flowering. The average values were considered for analysis.
- *The number of fruit clusters* The number of fruit clusters per plant were counted at physiological maturity from randomly selected five plants. The average values were considered for analysis.
- *Fruit length and diameter (cm)* Ten fruits of varying sizes (very large, large, medium, small and very small) were collected from each selected plant. The length and diameter of each fruit were measured using a digital caliper. The mean diameter of a fruit was obtained by adding the diameter of all the selected fruits and then dividing the sum by the number of selected fruits. The average values were used for the analysis.
- *Total number of fruit (ha*^*−1*^*)* The sum total number of fruits of successive harvests of pink to full-ripe stage, where dropped fruits were not considered at all.
- *Marketable fruit yield (kg ha*^*−1*^*)* Fruits whose diameter was > 3 cm and which were free of damage from the net plot area were considered marketable at each harvest using a sensitive balance. The total marketable fruit yield is the sum of successive harvests.
- *Unmarketable fruit yield (kg ha*^*−1*^*)* Fruits whose diameter were ≤ 3 cm and which were damaged by insect, diseases, sun burn, etc. from the net plot area were considered as unmarketable yield. The total unmarketable fruit yield is the sum of successive harvests.
- *Total fruit yield (kg ha*^*−1*^*)* This was obtained by adding average marketable and un marketable fruit yield of successive harvests.
- *Water use efficiency (kg m*^*−3*^*)* was the ratio of water used by tomato crops to produce yield to the amount of water supplied by irrigation and calculated by the following formula^47^
  $$WUE = \frac{Y}{ET}$$

where WUE is the water use efficiency, Y is the marketable fruit yield of tomato (kg), E_T_ is the evapotranspiration of the crop and supplied with irrigation water (m^−3^) for the crop.

### Statistical analysis

After the homogeneity test, the collected data were subjected to an analysis of variance (ANOVA) to evaluate the main and interaction effect of the factors (irrigation regime, and N rate) on the selected parameters using R studio during the first and second year. For the mean value, a split-split analysis of variance was employed. In this case, variety was considered as a main plot factor, irrigation regime was considered as a split plot factor, and nitrogen rate as a split-split plot factor. Whenever the treatment effects were significant, mean separation was performed using the Duncan’s multiple range test at a 5% level of significance. Correlation coefficients were calculated to study the associative relations among the measurement parameters, according to Gomez and Gomez^48^.

### Partial budget analysis

The economic analysis was done using a partial budget analysis, based on the procedures described by CIMMYT^49^. For this analysis, the variable cost of fertilizer (31.5 Ethiopian birr (ETB) kg^−1^) and labor (150 ETB person day^−1^) were considered at the time of planting and during other operations. The price of the tomato marketable fruit yield (8.5 ETB kg^−1^) was also taken into account. The return was calculated as total gross return minus the total variable cost. Net benefits and costs that varied between treatments were used to calculate the marginal rate of return on invested capital as we transitioned from a less costly to a costlier treatment. To draw farmers’ recommendations from the marginal analysis in this study, a 100% return on investments was used as a reasonable minimum acceptable rate of return.

## Results and discussion

### Pre-plant soil physicochemical properties (before planting)

The pre-sowing soil analysis result of selected soil-physicochemical properties of the experimental soil is presented in Table 3. The analysis results indicate that the soil's textural class is clay (Table 3). This type of soil is high moisture holding capacity. The mean pH of the soil was 7.2 which is in the neutral soil reaction^50^ and suitable for the production of most crops including tomatoes. The soil’s potential CEC (29.3 cmolc/kg) was in the high range^51^. According to Tadesse, et al.^52^ the soil's organic carbon and total nitrogen content were in the low range (Table 3). Therefore, the application of N-containing fertilizer is mandatory for increasing tomato yield. The exchangeable K content of the soil is rated as very high^53^. Similarly, Kassie et al.^54^ also reported that high K content in the study area. According to the rating developed by Olsen^43^ for the irrigated area, the soil available P content of the experimental soil is high. The high phosphorus availability in irrigated lowland Ethiopian soils is due to factors such as the soil’s geological origin, weathering processes, organic matter input, periodic flooding, microbial activity, and traditional agricultural practices^55^. These factors contribute to nutrient-rich soil, accelerated nutrient release, and efficient nutrient cycling, maintaining high phosphorus levels beneficial for crop growth. The same author classified the soil Olsen available P content of irrigated soil as < 12 mg kg^−1^ is low, 12–17 mg kg^−1^ marginal, 18–25 mg kg^−1^ is adequate and > 25 mg kg^−1^ is high. Similarly, other authors also reported that the high available phosphorus content of the study area with a mean value of 36.55 mg kg^−1^^56–58^.

**Table 3:** Soil physico-chemical properties of the experimental soil.

Sample #	Textural class	BD (g cm^3^)	pH (1:2.5)	CEC (cmo(+) kg^−1^)	Ex.K (cmo(+) kg^−1^)	Av.P (ppm)	OC (%)	OM (%)	T.N (%)
1	Clay (S = 20%, C = 44%, Si = 36%)	1.36	7.14	29.3	1.45	29.33	1.38	2.37	0.147
2	Clay (S = 20%, C = 46%, Si = 34%)	1.42	7.12		1.48	28.76	1.39	2.39	0.133
3	Clay (S = 16, C = 40, Si = 36)	1.41	7.1		1.23	29.68	1.36	2.35	0.133
Mean	Clay	1.39	7.12	29.3	1.39	29.3	1.38	2.37	0.14

*BD* bulk density, *CEC* cation exchange capacity, *Ex.K* exchangeable potassium, *Av.P* available phosphorus, *OM* organic matter, *T.N* total nitrogen, *S* sand, *C* clay, *Si* silt.

### Effect of variety and irrigation regime on mean growth, yield component and yield of tomato

The irrigation regime, including the type of irrigation system used, the level of deficit irrigation, and the soil moisture regime, can significantly influence the yield of tomato crops. By optimizing these factors, it’s possible to maximize yield while conserving water resources^59,60^. In our study, we found that variety significantly influenced most of the measured parameters. These included plant height (*P* ≤ 0.01), fruit length (*P* ≤ 0.001), fruit diameter (*P* ≤ 0.001), marketable fruit yield (*P* ≤ 0.05), unmarketable fruit yield (*P* ≤ 0.05), total yield (*P* ≤ 0.05), and WUE (*P* ≤ 0.01) (Table 6). The variety of a tomato plant can significantly affect its yield due to a combination of genetic factors, environmental adaptability, resistance to threats, soil condition, and planting techniques^61–63^. Therefore, choosing the right variety for the specific growing condition is crucial for maximizing yield. In general, Kochero variety demonstrated the highest yield across all levels of irrigation regimes and nitrogen fertilizer.

**Figure 3:**
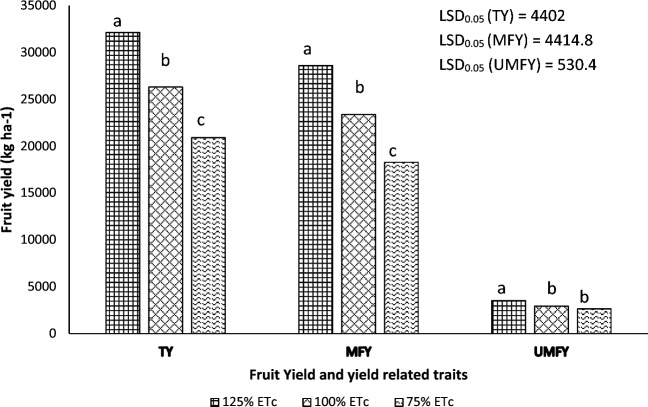
Fruit yield of tomato as influenced by different irrigation regime.

**Figure 4:**
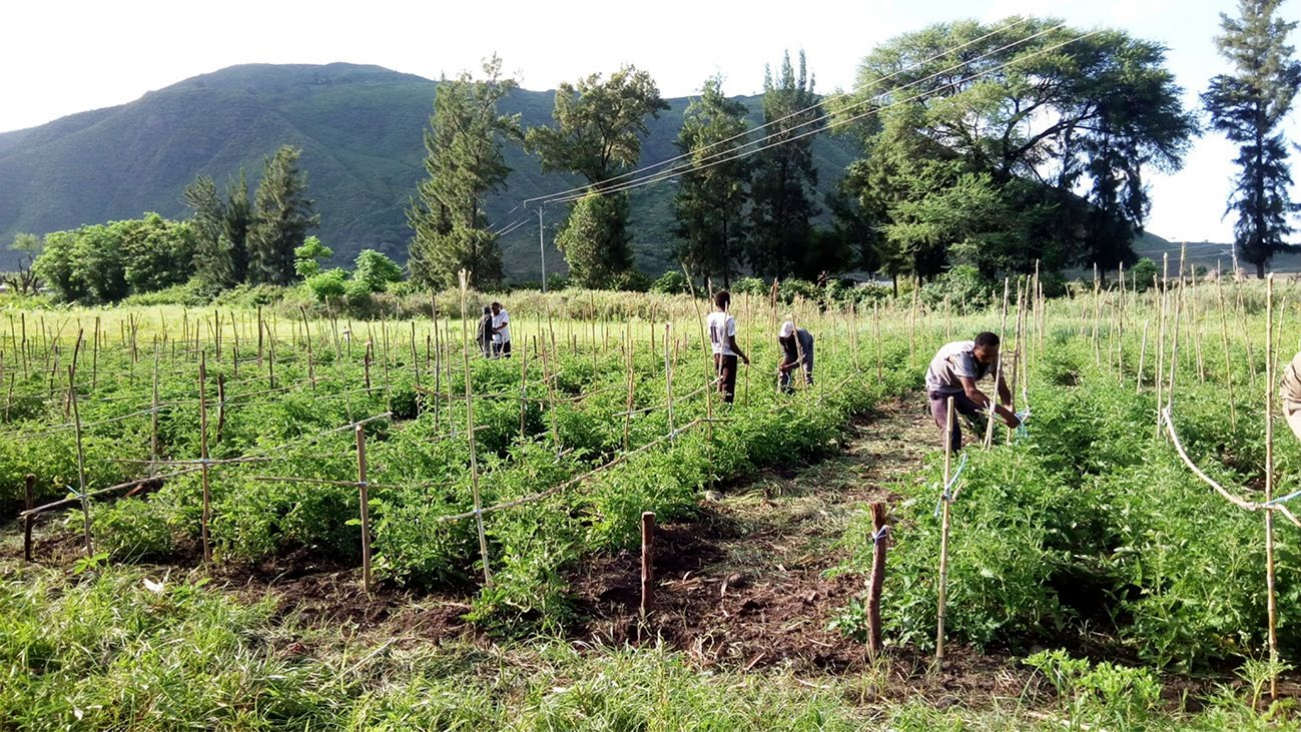
Field performance of tomato when stacking is prepared.

**Figure 5:**
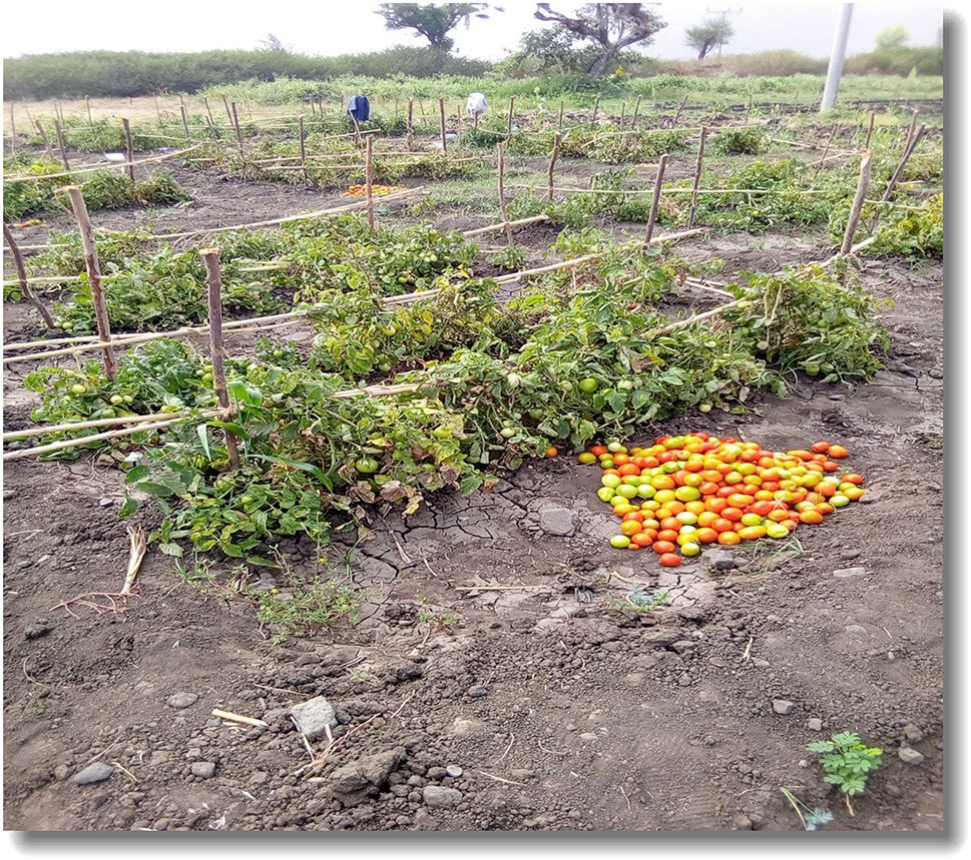
Field performance of tomato during the third harvesting stage.

Irrigation regime also significantly influenced marketable fruit yield (*P* ≤ 0.001), unmarketable fruit yield (*P* ≤ 0.001) and total yield (*P* ≤ 0.05) (Table 6). As the irrigation regime increased, so did the total, marketable, and unmarketable fruit yield of tomatoes (Fig. 3). Applying 125% ETc resulted in total yield advantage of 22% (5831 kg ha^−1^) and 54% (11,219 kg ha^−1^) compared to 100% ETc and 75% ETc, respectively. Similarly, the highest marketable fruit yield was obtained from the application of 125% ETc. This treatment increased the marketable fruit yield by 22% (5209 kg ha^−1^) and 56% (10,311 kg ha^−1^) compared to the application of 100% and 75% ETc, respectively (Fig. 3). The unmarketable fruit yield of tomatoes ranged from 3551 to 2643 kg ha^−1^ with the application of 125% ETc and 25% ETc, respectively. In between these, the application of 100% ETc resulted in unmarketable fruit yield of 2929 kg ha^−1^, respectively (Fig. 3). Edossa et al.^64^ also reported that the highest (82 t ha^−1^) and lowest (49 t ha^−1^) total yield of tomatoes were recorded from full irrigation and from 60% of full irrigation depth. The application of 80% of full irrigation resulted in total yield of 57 t ha^−1^. Indicating that tomato crop should be irrigated at full water requirement to get maximum fruit yield. Nevertheless, the irrigation regime generated by FAO’s CROPWAT model results in a lower yield of tomatoes, requiring 25% more irrigation water than the model suggests. This discrepancy could be due to the model’s reliance on local weather and soil data to calculate irrigation needs^65^. If these data are inaccurate or do not accurately reflect the actual conditions, the model’s may not lead to the highest possible yield. The models takes into account the sensitivity of different growth stages of the crop to water stress^66^. However, if the crop is particularly sensitive to water stress, providing extra water (125% of the recommended amount) might help the tomato to perform better and yield more^67^ The CROPWAT model also uses crop coefficient to adjust the reference evapotranspiration to the crop evapotranspiration. If this coefficient does not accurately represent the crop’s water use, the model’s irrigation recommendation may not be optimal. Nevertheless, Habtewold and Gelu^68^ reported that the higher marketable yield (36 t ha^−1^) and total yield (38 t ha^−1^) were obtained from 100% ETc, and the lowest marketable yield (19 t ha^−1^) and total yield (25 t ha^−1^) were obtained from a deficit level of 50% ETc in Arbaminch Zuria district in SNNPR region^68^. Bekele^69^ also reported that a treatment receiving 100% ETc irrigation level has a 7% and 15% yield increment as compared to 75% and 50% ETc irrigation levels, respectively. The same authors also reported that the application of 100% ETc level has a significant yield difference with 50% ETc level, but is at par with that of 75% ETc level. Other authors also reported that marketable yield of tomatoes was significantly affected by the amount of irrigation water applied^60,70–72^. Irrigation water application increased tomato yield by providing optimal moisture and nutrient availability for the plant growth and fruit development^73^ (Figs. 4, 5).

### Effect of nitrogen fertilizer on mean growth, yield component and yield of tomato

The effect of different rate of nitrogen fertilizer was signification plant height (*P* ≤ 0.05), the number of fruit cluster per plant (*P* ≤ 0.001), the number of unmarketable fruit (*P* ≤ 0.01), the total number of marketable fruit (*P* ≤ 0.01), the marketable fruit yield (*P* ≤ 0.001), the unmarketable fruit yield (*P* ≤ 0.05), total fruit yield (0.001), and WUE (*P* ≤ 0.01) (Table 6). The analysis indicated that the rate of N fertilizer increased, almost of the collected parameters result also increased progressively (Tables 4, 5). The highest number of fruit cluster per plant (12), the total number of fruit (849,124), and the marketable fruit yield (25,516 kg ha^−1^) were observed from the application of 138 kg N ha^−1^. This treatment increased the respective parameters by 23% (2), 75% (364,972), and 30% (5857 kg ha^−1^) compared with the lowest result recorded from nitrogen unfertilized plot (Table 4). The highest value of number of unmarketable fruit yield (83,444) was observed from application of 92 kg N ha^−1^. This treatment increased the respective parameters by 103% (40,970) compared with the lowest yield observed from nitrogen unfertilized plot (Table 4). Other studies have also shown that tomato yield tends to increase with nitrogen fertilizer in Ethiopia^74–77^. The positive effect of nitrogen on tomato yield and yield component is mainly because nitrogen is one of the most limiting nutrients, affecting plant growth and yield worldwide^78–80^. Nitrogen is also a crucial nutrient for the physiological and metabolic process in a tomato, as it increases the nitrogen uptake. Therefore, adequate nitrogen application in soil having low soil nitrogen increases tomato yield^81–83^. Tomato yield is adversely affected by poor soil fertility management, especially nitrogen, and lack of site-specific fertilizers recommendations^74,84–87^. Our results also confirmed that the soil of the experimental site is low in soil total nitrogen (Table 3). Therefore, the application of nitrogen containing fertilizer is mandatory for the test crop. The role of nitrogen application in increasing tomato yield is well-documented^74,76,88–92^ and as the rate of nitrogen fertilizer increased, the yield of tomato also increased^93^.

**Table 4:** Main effect nitrogen fertilizer on growth, yield related and yield of tomato.

N rate	PH	NFCPP	NUUMF	TNUF	MFY	UMFY
0	61.2^ab^	9.5^c^	39,925^b^	484,152^b^	19,659^b^	2395^b^
46	58.7^b^	10.4^bc^	74,166^a^	576,640^ab^	23,795^a^	3101^ab^
92	63.5^a^	11.4^ab^	83,444^a^	656,057^ab^	24,689^a^	3517^a^
138	64.5^a^	11.7^a^	80,895^a^	849,124^a^	25,516^a^	3151^ab^
LSD_0.05_	4.3	1.02	23,795	350,847	3221	842

*PH* plant height, *NFCPP* number of fruit cluster per plant, *NUUMF* number of un-marketable fruit, *TNUF* total number of fruit, *MFY* marketable fruit yield, *UMFY* un-marketable fruit yield.

**Table 5:** Interaction effect of irrigation depth and N on growth, yield related and yield of tomato.

N rate	NUUMF			TY (kg ha^−1^)			MFY (kg ha^−1^)			UMFY (kg ha^−1^)		
	75%ETc	100% ETc	125% ETc	75%ETc	100% ETc	125% ETc	75% ETc	100% ETc	125% ETc	75% ETc	100% ETc	125% ETc
0	34,791^e^	40,340^de^	44,643^de^	19,377^cd^	21,685^bcd^	25,099^bcd^	17,127^fg^	19,299^def^	22,551^cdef^	2250^c^	2386^c^	2548^bc^
46	58,898^cde^	58,553^cde^	105,048^ab^	26,436^bcd^	29,205^abc^	30,211^abc^	23,942^cde^	26,277^bc^	26,329^bc^	2493^bc^	2929^abc^	3882^ab^
92	118,986^a^	69,225^bcde^	62,122^cde^	16,552^d^	28,006^bcd^	40,062^a^	13,655^g^	24,510^bcd^	35,903^a^	2897^abc^	3495^abc^	4160^a^
138	99,073^abc^	67,440^bcde^	76,172^bcd^	21,316^bcd^	26,337^bcd^	33,184^ab^	18,385^efg^	23,429^cde^	29,571^b^	2931^abc^	2908^abc^	3613^abc^
LSD_0.05_	41,214			10,649			5579			1458		

*NUUMF* number of un-marketable fruit, *MFY* marketable fruit yield, *UMFY* un-marketable fruit yield, *TY* total yield.

### Interaction effect of irrigation regime and nitrogen rate on mean growth, yield component and yield of tomato

Combined over years, our study found that only the number of fruit cluster per plant, the number of unmarketable fruit, the marketable fruit yield, the total fruit yields, and WUE were significantly influenced by the interaction of irrigation regime and nitrogen rate (Table 6). Other parameters were not significantly influenced by this interaction. The highest number of unmarketable fruit yield (118,986) was recorded from the combined application of 75% ETc with 92 kg N ha^−1^, while the lowest number of unmarketable fruit (34,791) was recorded from 75% ETc with 0 kg N ha^−1^ (Table 5). Additionally, the highest (4160 kg ha^−1^) and lowest (4160 kg ha^−1^) yield of unmarketable fruit were recorded from the combined application of 125% ETc with 92 kg N ha^−1^ and 75% ETc with 0 kg N ha^−1^, respectively The highest (35,903 kg ha^−1^) and lowest (13,655 kg ha^−1^) yield of marketable fruits were recorded from the combined application of 125% ETc with 92 kg N ha^−1^ and 75% ETc with 92 kg N ha^−1^, respectively (Table 5). The result indicated that there was a consistent increase in yield with increasing irrigation regime across all levels of nitrogen nutrients. However, the yield increment was not consistent across all levels of the irrigation regime with application of nitrogen nutrients. This indicates that the yield of tomatoes is mainly determined by the application of irrigation water. Therefore, the best combination of these two factors for this study area was found to be 125% ETc and 192 kg N ha^−1^. Similarly, various scholars reported the effect of irrigation water and nutrient on tomato yield^64,75,94–97^. In vegetable crop production, nutrient, especially nitrogen and water management are related, and optimal management of one program requires good management of the other^98^. Du et al.^78^ reported that there was a significant interaction between the amount of irrigation water and applied nitrogen on tomato yield. Our result also confirmed that the interaction of the irrigation amount and nitrogen rate was significant. Tomato plants are sensitive to water stress^97^. Suboptimal application of nutrients and low soil fertility status, especially nitrogen and phosphorus also adversely affect tomato yield^99–101^. Our result also indicated that the proper combination of nitrogen fertilizer (92 kg N ha^−1^) and irrigation water (125% ETc) can increase the yield of marketable tomato by 163%. The interaction effect of the irrigation regime and nitrogen fertilizer affects tomato yield because different combinations of these factors influence soil moisture, nutrient availability, plant growth, and fruit quality of tomatoes. Both irrigation water and nitrogen fertilizer are essential for tomato production, but they need to be applied in appropriate amounts and forms to achieve optimal results^30,94^.

**Table 6:** Mean square values of mean plant growth and yield parameters of tomatoes as influenced by watering regimes and N rates.

	Var	Mean squares values with respective degrees of freedom in parenthesis										
		PH	NFCPP	FL	FD	NUMF(10^9^)	NUUMF (10^8^)	TNUF (10^9^)	MFY (10^6^)	UMFY (10^5^)	TY (10^6^)	WUE
Rep (3)	*1*	778**	23,2*	1.7^ns^	0.7^ns^	620^ns^	70^ns^	740^ns^	70	456*	95.6^ns^	1.28405^ns^
IRR (2)		105^ns^	3.3^ns^	2.1^ns^	2.2^ns^	330^ns^	13^ns^	300^ns^	800*	99.3**	967.9**	2.64198^ns^
Ea (6)		43	2.5	4.2	2.8	290	29	280	70	8.2	66	1.54434
N (3)		160^ns^	24.4***	0.6^ns^	3^ns^	480^ns^	98**	580^ns^	200**	52.8^ns^	218.8**	7.70147***
IRR*N (6)		24^ns^	4.8^ns^	8.4*	2.1^ns^	300^ns^	48*	310^ns^	100**	6.6^ns^	136.7**	8.11999***
Eb (27)		55	2.5	2.5	1.7	340	17	340	30	21.8	36	1.1944
CV (a)		12.7	22.4	0	0	121.1	81.8	109.8	26.1	37.4	22.5	25.65
CV (b)		17.1	17,2	0	0	118.4	79.4	108.9	22.2	37.9	20.6	22.56
Rep (3)	*2*	497.4**	52.5**	3.4^ns^	1.3^ns^	106*	66*	160*	190^ns^	38.1^ns^	246.6^ns^	5.6942^ns^
IRR (2)		23.4^ns^	1.1	4.2^ns^	4.4^ns^	12.2^ns^	18^ns^	22^ns^	12^ns^	9.5^ns^	13.4^ns^	10.0813*
Ea (6)		35.7	27.0	8.3	5.6	16.6	13	25	49	9.2	57	1.331
N (3)		141.3***	39.2***	1.3^ns^	6^ns^	71.9**	96***	130***	140***	36.2*	188.9***	4.4171***
IRR*N (6)		31.9^ns^	3.8*	16.8*	4.2^ns^	3.83^ns^	54^ns^	4.7^ns^	7.6^ns^	2.1^ns^	8.1^ns^	0.2961^ns^
Eb (27)		18.69	1.2	4.9	3.3	10.5	51	12	19	9.1	23	0.7707
CV (a)		8.8	15.2	5.8	6.3	28.8	48	30.5	38.8	47.5	37.9	36.26
CV (b)		6.3	10.3	4.4	4.9	22.9	29.8	21.4	24.3	47.4	24.1	27.59
Rep (3)		1037.3	30.9	0.7	0.27	824	93.2	986	90.3	60.7	128	3.411
Var (1)		4749.9**	0.95^ns^	17,677***	9221***	2010^ns^	43.5^ns^	1830^ns^	3810*	1344*	5375*	155.8*
Error A*B	3	54.9	40.1	0.7	0.27	444	27	419	238	63.2	308	9.015
IRR (2)		113.6^ns^	6.4^ns^	0.8^ns^	0.83^ns^	455^ns^	27.8^ns^	393^ns^	1090***	108*	1316***	61.595***
Var*IRR (2)		42.5^ns^	9.8^ns^	0.8^ns^	0.83^ns^	44^8ns^	52^ns^	393^ns^	829***	62.7*	978.4***	22.3***
Error A * B * C (12)		38.5	4.1	2.4	1.45	364	21.8	363	55.1	15.7	61	1.363
N (3)		212.8*	32.6***	0.3^ns^	1.21^ns^	636^ns^	130***	771^ns^	216***	70.4*	291.8***	8.8**
Var * N (3)		132.4^ns^	25.9***	0.3^ns^	1.21^ns^	267^ns^	33^ns^	219^ns^	229***	31.7^ns^	272.2***	9.87***
IRR * N (6)		41.6^ns^	5.5^ns^	2.5^ns^	0.67^ns^	396^ns^	56.7**	399^ns^	192***	7.1^ns^	195.3***	4.547*
Var * IRR * N (6)		114.5^ns^	6.9*	2.5^ns^	0.67^ns^	367^ns^	50.2**	367^ns^	129**	10.8^ns^	129.1**	3.76*
Error A * B * C * D (54)		58.4	2.5	1.3	0.94	349	15.3	352	33	17.9	38	1.514
Error (48)		17.2	1.5	1023	567	5.44	5.6	7.6	13.9	5.7	16	0.646
CV (Rep * Var)		11.96	58.79	5.21	4.29	116.5	74.66	100.9	65.82	82.66	66.31	64.82
CV (Rep * Var * IRR)		10.01	18.73	9.68	9.97	105.5	67.09	93.85	31.71	41.17	29.41	25.21
CV (Rep * Var * IRR * N)		12.33	14.77	7.18	8.03	103.3	56.19	92.55	24.54	44.02	23.35	26.56

*Var* variety, *Var 1* Kochero, *var 2* Weyno, *3* combined analysis, *N rate* nitrogen rate, *IRR* irrigation regime, *PH* plant height, *NFCPP* number of fruit cluster per plant, *FL* fruit length, *FD* fruit diameter, *NUMF* number of marketable fruit, *NUUMF* number of un-marketable fruit, *TNUF* total number of fruit, *MFY* marketable fruit yield, *UMFY* un-marketable fruit yield, *TY* total yield, *WUE* water use efficiency, *Ea* error term for the main plot, *Eb* error term for the sub plot. Number in parenthesis are degree of freedom.

### Partial budget analysis

According to the dominance analysis of the mean value; the application of 75% ETc with 92 kg N ha^−1^, 75% ETc with 138 kg N ha^−1^, 100% ETc with 0 kg N ha^−1^, 100% ETc with 138 kg N ha^−1^, 125% ETc with 0 kg N ha^−1^, 125% ETc with 46 kg N ha^−1^ and 125% with 138 kg N ha^−1^ were dominated by other treatments (Table 8) and were removed from further economic analysis. The result indicated that the highest (266,272 ETB) and lowest (91,567) net benefit were recorded from the combined application of 125% ETc with 92 kg N ha^−1^ and from 75% ETc with 92 kg N ha^−1^, respectively (Table 8). The Marginal Rate of Return (MRR) ranged from -1,140 with the combined application of 100% ETc with 92 kg N ha^−1^ to 3,890 with the combined application of 75% ETc with 92 kg N ha^−1^ (Table 8). Likewise, the combined application of 75% ETc with 46 kg N ha^−1^, 100% ETc with 46 kg N ha^−1^ and 125% ETc with 92 kg N ha^−1^ fulfilled the reasonable minimum acceptable rate of return (MRR) (100%). Therefore, the application of 125% ETc with 92 kg N ha^−1^ resulted in the highest net benefit as well as an acceptable rate of return (1240%) for the invested capital. The application of 75% ETc with 46 kg N ha^−1^ also resulted in the highest MRR (3890%) with a reasonable net benefit (180,458 ETB) and could be an alternative scenario if irrigation water is limited (Table 7).

**Table 7:** Partial budget analysis.

Treatment	Mean (MFY)	FGP	GB	CF	CL	TVC	NB	D	MB	MC	MRR (%)
75ETc*0 N	17,127	8.5	145,580	0	21,600	21,600	123,980	DM	0	0	
75% ETc*46 N	23,942	8.5	203,508	1450	21,600	23,050	180,458		56,477	1450	3890
75% ETc*92 N	13,655	8.5	116,067	2900	21,600	24,500	91,567	DM			
75% ETc*138 N	18,385	8.5	156,272	4350	21,600	25,950	130,322	DM			
100% ETc*0 N	19,299	8.5	164,049	0	28,800	28,800	135,249	DM			
100% ETc*46 N	26,277	8.5	223,352	1450	28,800	30,250	193,102		12,644	7200	160
100%ETc*92 N	24,510	8.5	208,338	2900	28,800	31,700	176,638		− 16,464	1450	− 1140
100%ETc*138 N	23,429	8.5	199,148	4350	28,800	33,150	165,998	DM			
125%ETc*0 N	22,551	8.5	191,679	0	36,000	36,000	155,679	DM			
125%ETc*46 N	26,329	8.5	223,798	1450	36,000	37,450	186,348	DM			
125%ETc*92 N	35,903	8.5	305,172	2900	36,000	38,900	266,272		89,634	7200	1240
125%ETc*138 N	29,571	8.5	251,354	4350	36,000	40,350	211,004	DM			

*FGP* farm gate price of tomato, *GB* gross benefit, *CF* cost of fertilizer, *CL* cost of labor, *TVC* total variable cost, *NB* net benefit, *D* dominance, *DM* dominated treatment, *MC* marginal cost, *MB* marginal benefit, *MRR* marginal rate of return.

### Water use efficiency

**Figure 6:**
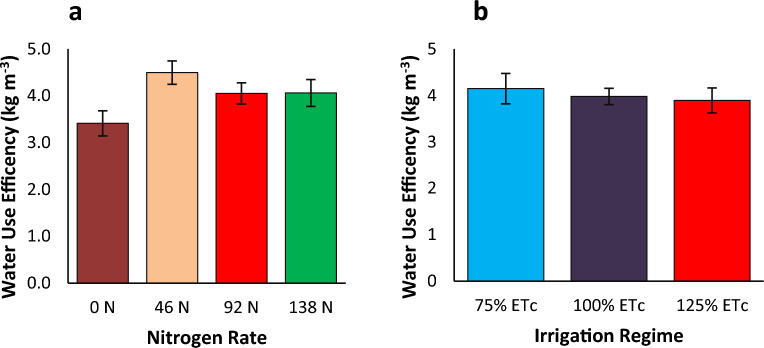
Water use efficiency of tomato as influenced by irrigation regime and nitrogen fertilizer application.

Water Use Efficiency (WUE) is defined as the total accumulated biomass per used unit of water applied^102^, indicating how effectively a crop uses water. WUE is a critical factor in agriculture, especially in areas where water is scarce. The combined analysis indicated that WUE was significantly influenced by the application of nitrogen, irrigation regime, and their interaction (Table 6). This suggests that optimizing both irrigation and nitrogen rate can improve WUE. The result showed that WUE increased with the application of nitrogen fertilizer (Fig. 6a). The application of 46, 92 and 138 kg N ha^−1^ increased WUE by 32% (1 kg m^−3^), 16% (0.6 kg m^−3^), and 19% (0.6 kg m^−3^), respectively, compared with the nitrogen-unfertilized control plot. Similarly, Cheng, et al.^103^ reported that water use efficiency of tomato is improved by 21% with the application of nitrogen fertilizer. This is mainly because the application of nitrogen fertilizer can affect the water use efficiency of tomato by influencing the water uptake, transpiration, and evaporation processes with optimal rate and timing of fertilizer^104^. The observed increase in WUE with nitrogen fertilizer application sheds light on the importance of nitrogen management in agricultural sustainability. The positive impact on WUE at different nitrogen application rates demonstrates the potential for optimizing nitrogen use to enhance resource efficiency^105^. Moreover, the specific magnitudes of WUE improvement associated with different nitrogen levels provide practical insights for farmers and policymakers seeking to maximize productivity while minimizing environmental impact. Irrigation levels that are too high often result in surplus water runoff and evaporation losses, particularly in systems that are not optimally managed. By curbing the volume of irrigation, farmers can limit these losses, ensuring a greater amount of water is available for plant absorption and reducing overall water wastage. Over-irrigation has the potential to wash nutrients out of the soil, thereby reducing their accessibility to plants^106^. By judiciously managing water application, farmers can sustain optimal nutrient levels in the root zone, fostering superior nutrient absorption by tomato plants and maximizing their growth potential. Overwatering can foster conditions conducive to the proliferation of waterborne diseases and pests, such as root rot and fungal infections^107^. By embracing a more restrained irrigation strategy, farmers can alleviate these risks, resulting in healthier plants and increased yields.

**Figure 7:**
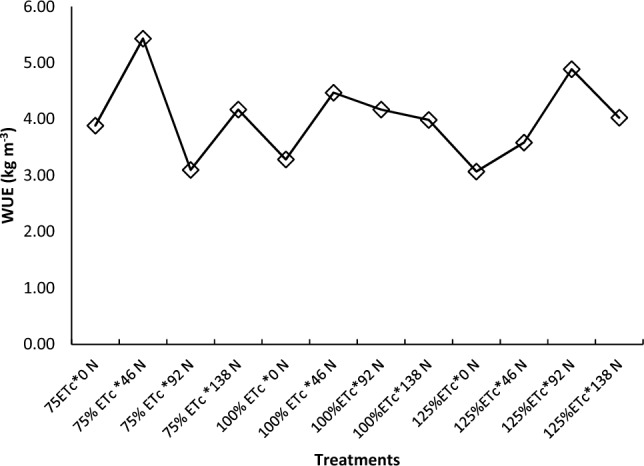
Water use efficiency of tomato as influenced by the interaction of irrigation regime and nitrogen fertilizer application.

In our study, there was inverse relation between irrigation regime and WUE (Fig. 6b). Generally, a lower irrigation regime had higher WUE. This might be attributed to the fact that over irrigation can lead to water shortage. This excess water does not contribute to plant growth or yield but is lost through runoff or deep percolation^60^. As a result, the WUE, which is the ratio of yield to water used, decreased as irrigation depth increase beyond the optimal rate. Nutrient leaching due to over-irrigation can also cause nutrient leaching, where essential nutrients are washed away from the root zone. This can reduce plant growth and yield, further decreasing WUE^60^. Deficit irrigation can induce water stress in plant, affecting their physiological process. For instance, when the plant consumption of irrigation water reduced from 100 to 50%, stomatal conductance of tomato decreased by 45%^108^. This can lead to reduced WUE. In our study, the highest WUE recorded from 75%% ETc increased WUE by 4%, and 7% compared with 100% ETc and 125% ETc, respectively. The observed increase in WUE with reduced irrigation levels highlights the potential benefits of implementing deficit irrigation strategies in agricultural systems. By optimizing water management practices, farmers can achieve higher crop yields while conserving water resources and promoting sustainable agriculture^109^. However, it's essential to acknowledge that the optimal level of deficit irrigation may vary depending on factors such as crop type, soil characteristics, climate conditions, and management practices^110,111^. Further research is needed to determine the specific thresholds and conditions under which deficit irrigation can maximize WUE without compromising crop productivity. The analysis of variance showed that the highest WUE (5.4 kg m^−3^), recorded from 75% ETc with 46 kg N ha^−1^, increased WUE by 77% (2.4 kg m^−3^) compared with the lowest WUE (2.3 kg m^−3^) recorded from 125% ETc with 0 kg N ha^−1^ (Fig. 7). This indicates that WUE can be improved by decreasing water application to the crop.

At the same nitrogen rate (for instance, at 46 kg N ha^−1^), the application of 75% ETc increased WUE by 21% (1 kg m^−3^), and 52% (1.8 kg m^−3^) compared with 100% and 125% ETc, respectively (Fig. 7). Under conditions of water scarcity, the optimal approach involves applying 75% ETc with 46 kg N ha^−1^ due to its superior MRR and water conservation advantages. This allows for an additional irrigation potential of 0.25 and 0.5 ha of land compared to applying 100% ETc and 125% ETc, respectively. Similarly, Dong et al.^112^ reported that the application of 50% of the estimated crop water requirement (CWR) of tomato resulted in the highest WUE and marketable yield in Ethiopia. Other study also indicated that tomatoes crop irrigated at 80% reference crop evapotranspiration (ET0) and supplied with 180 kg N ha^−1^ under drip fertigation had a satisfactory fruit yield of tomatoes^104^. This suggests that optimizing both irrigation and N rates can improve WUE. This mainly because different regimes can have different impacts on the soil moisture, nutrient availability, plant growth, and fruit quality of tomatoes^73^. Improving the water use efficiency of tomatoes is important for several reasons. First, it can help save water and reduce the pressure on water resources, especially in areas where water is scarce or expensive. Second, it can improve the yield and quality of tomatoes by avoiding water stress or excess, which can affect the growth, development, and physiology of the plant. Third, it can enhance the profitability and sustainability of tomato production by reducing the costs and environmental impacts of irrigation^60,113^. These results underscore the need for integrated approaches to agricultural management that consider multiple factors simultaneously. By strategically adjusting nitrogen application and irrigation practices based on crop needs and environmental conditions, farmers can strive for greater WUE and overall sustainability in agricultural production systems. Additionally, further research could delve deeper into the underlying mechanisms driving the observed responses, facilitating more targeted management strategies in the future.

### Correlation among parameters

The correlation analysis indicated that WUE was significantly and positively correlated with total fruit yield (R = 0.6852), marketable fruit yield (R = 0.7022), and the number of fruit cluster per plant (R = 0.3821) (Table 8). The plant height of tomato was significantly and positively correlated with the number of fruit cluster per plant (R = 0.4808). There was a negative correlation between plant height and lateral branch length (R = − 0.4592) (Table 8). The number of fruit cluster per plant was found to be significantly correlated with the number of marketable fruit (R = 0.348), the number of unmarketable fruit (R = 0.3024), the total number of fruit (R = 0.3666), the total fruit yield (R = 0.5016), marketable fruit yield (R = 0.4704), and the unmarketable fruit yield (R = 0.4683).

**Table 8:** Correlation among the collected parameters.

	WUE	PH	LBL	NFCPP	FL	FD	NUMF	NUUMF	TNUF	MFY	UMFY	TY
WUE	1	0.1402^ns^	− 0.0581^ns^	0.3821***	− 0.1053^ns^	− 0.2201^ns^	0.2599^ns^	− 0.1348^ns^	0.2424^ns^	0.7022***	0.2537^ns^	0.6852***
PH	0.1402^ns^	1	− 0.4592***	0.4808***	− 0.1^ns^	− 0.1625^ns^	0.1303^ns^	0.1183^ns^	0.1377^ns^	0.0418^ns^	0.1832^ns^	0.0648^ns^
LBL	− 0.0581^ns^	− 0.4592***	1	− 0.2592^ns^	0.1119^ns^	− 0.0322^ns^	− 0.1249^ns^	− 0.1611^ns^	− 0.1361^ns^	− 0.0102^ns^	0.0092^ns^	− 0.0082^ns^
NFCPP	0.3821***	0.4808***	− 0.2592^ns^	1	0.0476^ns^	− 0.0348^ns^	0.348*	0.3024*	0.3666*	0.4704***	0.4683***	0.5016***
FL	− 0.1053^ns^	− 0.1^ns^	0.1119^ns^	0.0476^ns^	1	0.2493^ns^	− 0.0425^ns^	0.1485^ns^	− 0.0286^ns^	0.0283^ns^	0.0445^ns^	0.0325^ns^
FD	− 0.2201^ns^	− 0.1625^ns^	− 0.0322^ns^	− 0.0348^ns^	0.2493^ns^	1	− 0.1982^ns^	− 0.0661^ns^	− 0.1996^ns^	− 0.0904^ns^	− 0.0469^ns^	− 0.0903^ns^
NUMF	0.2599^ns^	0.1303^ns^	− 0.1249^ns^	0.348*	− 0.0425^ns^	− 0.1982^ns^	1	0.2135^ns^	0.9964***	0.2298^ns^	0.2052^ns^	0.2417^ns^
NUUMF	− 0.1348^ns^	0.1183^ns^	− 0.1611^ns^	0.3024*	0.1485^ns^	− 0.0661^ns^	0.2135^ns^	1	0.2959*	− 0.0383^ns^	0.2852*	0.0054^ns^
TNUF	0.2424^ns^	0.1377^ns^	− 0.1361^ns^	0.3666*	− 0.0286^ns^	− 0.1996^ns^	0.9964***	0.2959*	1	0.2214^ns^	0.2255^ns^	0.2368^ns^
MFY	0.7022***	0.0418^ns^	− 0.0102^ns^	0.4704***	0.0283^ns^	− 0.0904^ns^	0.2298^ns^	− 0.0383^ns^	0.2214^ns^	1	0.4759***	0.9921^ns^
UMFY	0.2537^ns^	0.1832^ns^	0.0092^ns^	0.4683***	0.0445^ns^	− 0.0469^ns^	0.2052^ns^	0.2852*	0.2255^ns^	0.4759***	1	0.5826***
TY	0.6852***	0.0648^ns^	− 0.0082^ns^	0.5016***	0.0325^ns^	− 0.0903^ns^	0.2417^ns^	0.0054^ns^	0.2368^ns^	0.9921^ns^	0.5826***	1

*PH* plant height, *NFCPP* number of fruit cluster per plant, *FL* fruit length, *FD* fruit diameter, *NUMF* number of marketable fruit, *NUUMF* number of un-marketable fruit, *TNUF* total number of fruit, *MFY* marketable fruit yield, *UMFY* un-marketable fruit yield, *TY* total yield.

**Significant at the 1% level.

*Significant at the 5% level.

## Conclusion

In tomato farming, the interplay between nutrient, particularly nitrogen, and water management is crucial. Our study found that both the irrigation regime and nitrogen application significantly impact tomato yield and water use efficiency (WUE). Optimal results were achieved with a higher irrigation level (125% ETc) and optimal nitrogen application (92 kg N ha^−1^), yielding the highest marketable fruit yield and net return. However, in water-limited conditions, a reduced irrigation level (75% ETc) with a lower nitrogen application (46 kg N ha^−1^) provided the highest WUE. Thus, we recommend the former for tomato production in areas with sufficient water, and the latter as a viable alternative when irrigation water is scarce.

## Data Availability

The data sets used during the current study are available from the corresponding author on reasonable request.

## Abbreviations

ANOVA: Analysis of variance
ETc: Potential evapotranspiration from the crop
CIMMYT: International Maize and Wheat Improvement Center
ETB: Ethiopian Birr
FAO: Food and agricultural organization of the united nation
